# Neurophysiological insights in dystonia and its response to deep brain stimulation treatment

**DOI:** 10.1007/s00221-020-05833-8

**Published:** 2020-07-07

**Authors:** Stephen Tisch, Patricia Limousin

**Affiliations:** 1 School of Medicine, University of New South Wales, Sydney, Australia; 2 Department of Neurology, St Vincent’s Hospital, Sydney, Australia; 3 Department of Clinical and Movement Neurosciences, UCL Queen Square Institute of Neurology, 33 Queen Square, 2nd floor, Box 146, London, WC1N 3BG UK

**Keywords:** Dystonia, Neurophysiology, Gpi DBS

## Abstract

Dystonia is a movement disorder characterised by involuntary muscle contractions resulting in abnormal movements, postures and tremor. The pathophysiology of dystonia is not fully understood but loss of neuronal inhibition, excessive sensorimotor plasticity and defective sensory processing are thought to contribute to network dysfunction underlying the disorder. Neurophysiology studies have been important in furthering our understanding of dystonia and have provided insights into the mechanism of effective dystonia treatment with pallidal deep brain stimulation. In this article we review neurophysiology studies in dystonia and its treatment with Deep Brain Stimulation, including Transcranial magnetic stimulation studies, studies of reflexes and sensory processing, and oscillatory activity recordings including local field potentials, micro-recordings, EEG and evoked potentials.

## Introduction

Dystonia is defined by “sustained or intermittent muscle contractions that lead to abnormal movements, postures, or both” (Fahn 1988). The most recent consensus definition of dystonia includes tremor and highlights that dystonic movements are “typically patterned, twisting, and may be tremulous, initiated or worsened by voluntary action and associated with overflow muscle activation” (Albanese et al. 2013). There are many different types of dystonia and causes; the current classification system uses dual axis of clinical features such as age of onset, body distribution and underlying cause such as neuropathology or gene (Albanese et al. 2013).

Although the pathophysiology of dystonia is not fully understood, the loss of intracortical inhibition, increased cortical plasticity and abnormal sensorimotor integration are known to be involved in dystonia.

Deep brain stimulation of the globus pallidus interna (GPi-DBS) is considered effective and safe in refractory dystonia (Krauss et al. 1999; Coubes et al. 2000; Vidailhet et al. 2005; Kupsch et al. 2006; Volkmann et al. 2012, 2014). The time course of the effect is gradual but the mobile components tend to respond quickly and the tonic elements take longer (Chung and Huh 2016). In addition, different causes of dystonia can have different outcomes after DBS (Tisch 2018). GPi is the most commonly used target but some patients have also been implanted in the subthalamic nucleus (STN) and the thalamus.

DBS has also brought the opportunity to broaden research options in dystonia by allowing the record activity as well as measuring physiological changes in parallel to improvement. Here we review the neurophysiology of dystonia and its response to DBS.

## Transcranial magnetic stimulation (TMS)

The development of TMS by Barker in 1985 (Barker et al. 1985) and its refinement as a viable tool for in vivo assessment of motor cortex and cortico-motor pathways (Rothwell 1991) heralded an important chapter in an improved pathophysiological understanding of dystonia. Concepts of decreased excitability of inhibitory circuits within the brain and spinal cord underlying dystonia, derived from studies of H-reflex reciprocal inhibition (Nakashima et al. 1989) and blink reflexes (Berardelli et al. 1985) led to initial TMS studies evaluating cortical excitability in dystonia. The first TMS study in dystonia was performed by Ridding and Rothwell in 1995 and compared focal hand dystonia patients with healthy subjects and showed reduced short latency intracortical inhibition (SICI) at rest, interpreted as evidence for decreased excitability within intracortical inhibitory circuits (Ridding et al. 1995). An additional important finding of this study was that defective cortical inhibition was present bilaterally despite dystonia being present only unilaterally, providing evidence of distributed pathophysiological abnormalities in brain regions unaffected by dystonia, a finding replicated in other studies leading to the important concept of endophenotypic abnormalities acting as a substrate upon which environmental factors may operate to produce dystonia (Meunier et al. 2001). Further TMS studies demonstrated that reduced intracortical inhibition was present not only at rest but also preceding (Gilio et al. 2003) and during voluntary movement in focal hand dystonia patients (Chen et al. 1997a, b; Stinear and Byblow 2004). Similar reductions in cortical inhibition were found in segmental and generalised forms of dystonia (Rona et al. 1998) and non-manifesting carriers of TOR1A/DYT1 (Edwards et al. 2006), further supporting the notion of abnormal endophenotypes in dystonia. Using TMS techniques it has been demonstrated that patients with musician’s dystonia have greater reductions in SICI during hand muscle vibration stimuli than patients with writer’s cramp suggesting sensory input playing a greater role in musician’s dystonia (Rosenkranz et al. 2005). Collectively, TMS studies have provided evidence of reduced excitability of inhibitory circuits within the motor cortex providing further support for the loss of inhibition model of dystonia where action selection and topographic motor specificity are compromised leading to unwanted movements and co-contraction (Mink 1996; Hallet 2004).

TMS studies have also elucidated important interactions between pre-motor cortex and primary motor cortex in dystonia. In patients with writer’s cramp, low-frequency inhibitory TMS over the premotor cortex results in prolongation of the primary motor cortex silent period reflecting reversal of abnormally reduced motor cortex inhibition and a corresponding improvement in handwriting performance (Murase et al. 2005). Abnormally increased inhibition of the primary motor cortex by the premotor cortex has been demonstrated in patients with writer’s cramp at rest and during movement using a paired pulse TMS paradigm. In the same study, premotor cortex single pulse TMS preceding a manual choice reaction time task, reduced error rates without altering reaction time. The inhibition exerted by premotor cortex on primary motor cortex is likely supra-spinal as reflected in unchanged upper limb H-reflex amplitudes, and has been interpreted as compensatory in response to underlying disinhibition of the primary motor cortex in dystonia (Richardson et al. 2014).

A significant development in TMS studies for dystonia appeared with techniques allowing the elicitation of short-term cortical plasticity effects resembling long- term potentiation (LTP) and long-term depression (LTD). Low-frequency rTMS (repetitive TMS) around 1 Hz leads to sustained decreases in motor cortex excitability (Chen et al. 1997a; Touge et al. 2001), higher frequency 5–25 Hz rTMS produces sustained increases in cortical excitability (Peinemann et al. 2004; Khedr et al. 2007), and pulsatile 50 Hz theta burst rTMS can efficiently induce either sustained increases or decreases in motor cortex excitability (Huang et al. 2005).

Motor cortex plasticity can also be generated using combined TMS and median nerve stimulation with 25 ms interval between stimuli, so called paired associative stimulation (PAS) which allows topographically restricted increase in motor cortex excitability that can be blocked by NMDA antagonists suggesting LTP-like mechanisms (Stefan et al. 2000, 2002). With shorter interstimulus intervals LTD like effects from PAS have also been demonstrated (Wolters et al. 2003). Patients with focal dystonia display increased motor cortex plasticity to TMS PAS with reduced cortico-motor topographic specificity (Quartarone et al. 2003; Weise et al. 2006), providing evidence that altered sensorimotor cortical plasticity may play an important role in dystonia. Motor cortex plasticity following TMS PAS is normal in patients with functional dystonia and increased in organic dystonia patients (Quartarone et al. 2009), whereas cortical and spinal disinhibition is equal in both conditions (Espay et al. 2006; Avanzino et al. 2008), suggesting that motor cortex plasticity changes may be a more fundamental feature of organic dystonia. The role of the cerebellum in modifying motor cortex plasticity in dystonia has been evaluated using TMS cerebellar cortex stimulation combined with PAS during hand–eye coordination task, which found that patients with writer’s cramp have defective cerebellar inhibition of motor cortex plasticity (Hubsch et al. 2013). Increased brain plasticity in dystonia has also been demonstrated using TMS protocols other than PAS including low-frequency 1 Hz (Siebner et al. 2003; Baumer et al. 2007) and theta burst rTMS, where patients with cervical and segmental dystonia showed greater and longer lasting motor cortex inhibition than healthy subjects or non-manifesting TOR1A/DYT1 carriers (Edwards et al. 2006).

**Fig. 1:**
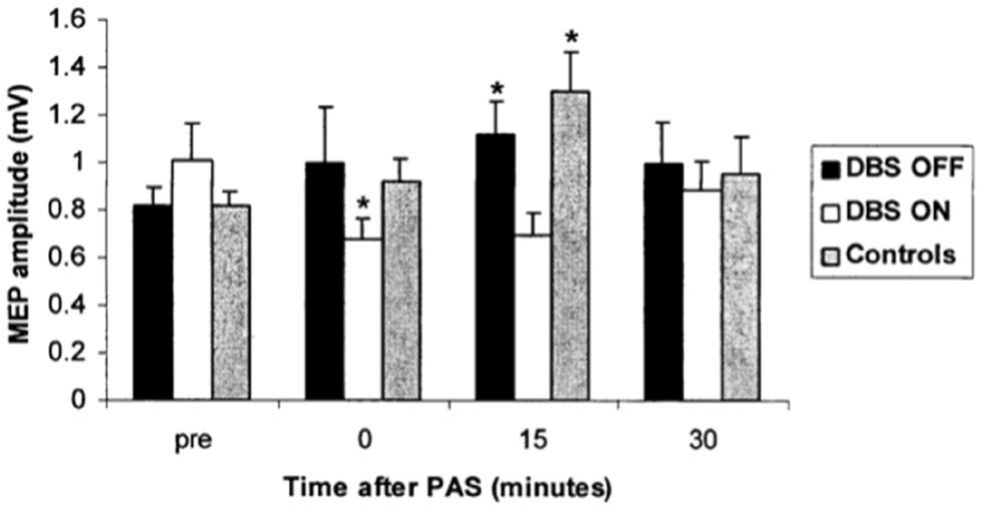
Effect of PAS on resting MEP amplitude with GPi DBS ON and OFF in dystonia patients. Note that DBS ON abolishes excitatory post-PAS plasticity (decrease in MEP amplitude), whereas DBS OFF and control subjects show preserved PAS response

The emergence of GPi DBS stimulation as an effective treatment for dystonia (Krauss et al. 1999; Coubes et al. 2000; Vidailhet et al. 2005) generated interest as to the underlying physiological mechanisms of improvement in dystonia after GPi DBS (Tisch et al. 2007a) and provided new opportunities to assess dystonia pathophysiology. The Queen Square group, including Prof John Rothwell, was among the first to utilize TMS techniques to evaluate the physiological effects of GPi DBS in dystonia. The safety of TMS in patients with implanted DBS systems had been established in a previous study which evaluated TMS cortical excitability measures in patients with GPi DBS ON and OFF stimulation and demonstrated a reversible reduction in motor cortex excitability with GPi DBS OFF with no effects on intracortical inhibition (Kuhn et al 2003). The first study assessing effects of GPi DBS on motor cortex plasticity using TMS included ten patients with idiopathic isolated generalised dystonia, some TOR1A/DYT1 positive with stable significant improvement following effective GPi DBS evaluated using TMS PAS with DBS ON and OFF in separate sessions. With GPi DBS OFF patients showed similar degree of motor cortex plasticity to PAS as healthy controls; however, with DBS ON, excitatory motor cortex plasticity was abolished and shifted to cortical inhibition, which correlated with the degree of clinical improvement from DBS (Tisch et al. 2007b). The study provided evidence of reversal of abnormally excessive motor cortex plasticity as a possible mechanism of action of GPi DBS for dystonia (see Fig. 1). The same group went on to study longitudinal changes in motor cortex excitability and plasticity using TMS PAS in a cohort of dystonia patients before and after DBS and found that SICI was reduced and plasticity increased in dystonia patients prior to DBS. One month after DBS, TMS PAS motor cortex plasticity was abolished but gradually increased towards normal levels at 3 and 6 months, while SICI improved gradually over the same time course mirroring progressive improvement in clinical symptoms (Ruge et al. 2011a). The progressive time-course of improvement in dystonia symptoms after GPi DBS is well described (Yianni et al. 2003; Vidailhet et al. 2005; Tisch et al. 2006a, b) and the finding of longitudinal changes in experimentally induced motor cortex plasticity suggests a potential mechanistic role. How might GPi DBS decrease excessive motor cortex plasticity in dystonia? It is known GPi DBS reduces ipsilateral excessive cortical activation in premotor and primary motor areas likely through enhanced thalamocortical inhibition (Kumar et al. 1999; Detante et al. 2004). A further mechanism may be increased background activity from GPi DBS, which may interfere with plasticity formation as demonstrated when anodal direct current stimulation is applied during PAS (Nitsche et al. 2007). Some further clues come from an interesting study, which evaluated the time-course of TMS PAS motor cortex plasticity in long-term GPi dystonia patients before and after switching off the DBS for 2 days (Ruge et al. 2011b). In keeping with previous studies PAS plasticity was almost absent and SICI was reduced with DBS ON. With DBS OFF for 2 days there was no change in SICI or plasticity at a group level, however there was a strong correlation between the amount of PAS plasticity ON DBS and the retention of clinical benefit after stopping DBS. These results suggest that many years of GPi DBS results in long-term changes in motor cortex plasticity underlying clinical benefit and that individual variation in plasticity profiles may dictate the extent to which clinical benefits persist.

In summary, TMS has delivered important insights in dystonia pathophysiology and mechanisms of action of GPi DBS and the contribution of Professor John Rothwell to this field of study merits special acknowledgement.

## EMG, reflex studies and sensory processing in dystonia

Traditional perspectives of dystonia as primarily due to abnormal basal ganglia activity have evolved to current the view of dystonia as a network disorder operating at all levels of the nervous system including cerebral cortex, thalamus, basal ganglia, cerebellum, brain stem and spinal cord (Tisch 2018). Important clinical hallmarks of dystonia including co-contraction and overflow of muscle activity, task and position specificity and sensory tricks (geste antagoniste), all recognized clinically for more than a century, are mediated by abnormal dystonia networks in which abnormal reflex activity to sensory inputs and sensory misprocessing play an important role. This section focuses on neurophysiological studies into these aspects.

### EMG studies in dystonia

Early studies using surface electromyography (EMG) demonstrated co-contraction of agonist and antagonist muscles in a task-specific manner during handwriting in patients with writer’s cramp (Rothwell et al. 1983). Patients with arm dystonia in the setting of idiopathic isolated segmental and generalized dystonia show abnormal co-contraction, muscle overflow activity and prolonged burst duration during voluntary elbow movements (Van der Kamp et al. 1989). Abnormal co-contraction in dystonia differs from voluntary co-contraction by virtue of abnormally coherent, synchronized motor unit activity driven by central descending input to motor neurons (Farmer et al. 1998). Reductions in low-frequency (4–12 Hz) intermuscular EMG coherence activity occur after effective GPi DBS and partially correlate with clinical improvement (Doldersum et al. 2019). EMG studies in dystonia have also characterized myoclonic jerks in myoclonus dystonia (Obeso et al. 1983; Li et al. 2008) and rhythmic bursting activity corresponding to dystonic tremors (Jedynak et al. 1991).

### H-reflex and blink reflex in dystonia

Reflex excitability is altered in dystonia and in general a pattern of reduced excitability of inhibitory circuits resulting in reduced inhibition has been observed. At the spinal cord level the H-reflex, the neurophysiological equivalent of a tendon jerk, is modulated by disynaptic and presynaptic inhibition by 1a afferents of the antagonist muscle, measured as H-reflex reciprocal inhibition, which is turn modified by excitability of local spinal cord circuits and descending input. H-reflex reciprocal inhibition is decreased in focal, segmental and generalized dystonia (Nakashima et al. 1989; Panizza et al. 1989, 1990) even in unaffected limbs (Deuschl et al. 1992; Chen et al. 1995) consistent with an endophenotypic abnormality. Abnormally reduced presynaptic phase of H-reflex reciprocal inhibition is reversed after botulinum toxin therapy (Priori et al. 1995) likely due to denervation of intrafusal fibres and reduced muscle spindle afference (Giladi 1997; Rosales and Dressler 2010).

The blink reflex is characterised by an early oligosynaptic R1 and later polysynaptic R2 components. The R2 component displays paired pulse inhibition with repeated stimuli (Kimura and Harada 1976) mediated by brainstem inhibitory interneurons, themselves under control by projections from cerebral cortex, thalamus and basal ganglia (Berardelli et al. 1983). Blink reflexes thus provide a useful measure of brainstem excitability and indirectly probe basal ganglia output. Blink reflex R2 inhibition is abnormally decreased in patients with cranial (Berardelli et al. 1985), cervical (Tolosa et al. 1988), segmental and generalised dystonia including those without blepharospasm (Nakashima et al. 1990) and is present equally in manifesting and non-manifesting TOR1A/DYT1 carriers (Fong et al. 2016). Unlike H-reflex reciprocal inhibition, blink reflex inhibition is normal in functional blepharospasm, and may assist in differentiating it from organic blepharospasm (Schwingenschuh et al. 2011).

### Effects of GPi DBS on reflex circuits

**Fig. 2:**
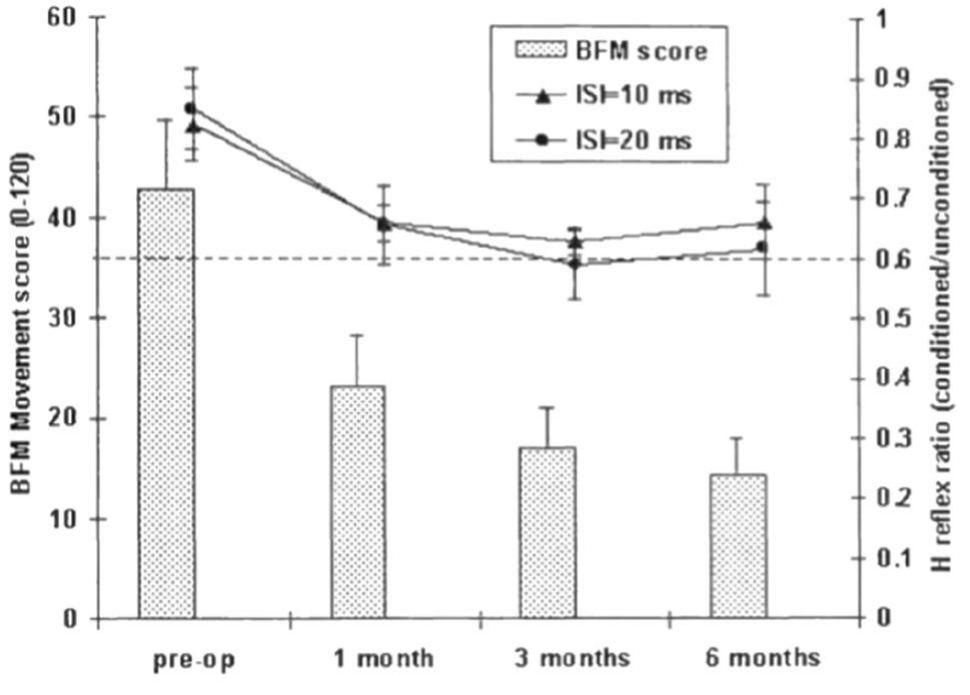
Time-course of changes in presynaptic phase of H-reflex reciprocal inhibition (RI) after GPi DBS and clinical improvement. A line is superimposed at 0.6, which represents a normal level of RI. Clinical improvement and changes in RI correlate and follow a logarithmic curve

GPi DBS restores abnormally reduced H-reflex reciprocal inhibition and blink reflex R2 inhibition in patients with generalized dystonia in a progressive time-course correlating with clinical improvement (see Fig. 2), indicating gradual normalisation of disinhibition within spinal and brainstem circuits as a marker or mechanism for clinical improvement ().

### Sensory processing in dystonia

Sensory involvement in dystonia is evident clinically by potent temporary reduction in dystonic symptoms with specific sensory tricks (Leis et al. 1992), the finding that lesions of the sensory system both central (Lehericy et al. 2001) and peripheral (Jankovic and Linden 1988) may result in acquired dystonia and the presence of sensory symptoms among dystonia sufferers (Ghika et al. 1993; Stamelou et al. 2012). Neurophysiology studies have provided further insights into wide-ranging abnormalities of sensory processing in dystonia.

Peripheral sensory input in the form of tonic vibration of the muscle tendon in patients with writer’s cramp worsens dystonic symptoms, while intramuscular injection of lignocaine improves dystonia, both effects likely mediated by alteration in muscle spindle afference, as anaesthetic inhibition of Ia fibres which mediate the tonic vibration response may help to compensate for loss of presynaptic inhibition in dystonia (Kaji et al. 1995).

In dystonia there is abnormal summation of cortical sensory evoked potentials in response to peripheral nerve stimulation (Tinazzi et al. 2000) and enlarged, dedifferentiated somatosensory receptive fields in thalamic receiving neurons such that the receptive field overflows from the sensory Vc thalamic nucleus to the cerebellar outflow-motor cortex relay Vim nucleus (Lenz et al. 1999). As mentioned, the hand representation within the somatosensory cortex is also distorted in dystonia including the asymptomatic side (Meunier et al. 2001). In patients with focal hand dystonia, tactile spatial discrimination is reduced (Bara-Jimenez et al. 2000a) and temporal discrimination of tactile and visuo-tactile stimuli is abnormally prolonged (Bara-Jimenez et al. 2000b; Fiorio et al. 2003). Sensory temporal discrimination is also prolonged non-manifesting TOR1A/DYT1 carriers (Fiorio et al. 2007) and unaffected first-degree relatives of dystonia sufferers in a frequency suggestive of an incompletely penetrant dominant gene (Kimmich et al. 2011), supporting an endophenotypic role for abnormal temporal discrimination in dystonia (Conte et al. 2017). A study using combined sensory temporal discrimination testing and evoked potentials indicated that prolonged tactile sensory discrimination thresholds are the result of reduced excitability of inhibitory circuits within the primary somatosensory cortex (Antelmi et al. 2017). Abnormally prolonged sensory temporal discrimination thresholds in dystonia can be further worsened by high-frequency peripheral sensory stimulation, suggesting abnormal plasticity with the primary somatosensory cortex (Erro et al. 2018). Perhaps surprisingly, in idiopathic isolated generalized dystonia tactile spatial sensory discrimination is normal (Molloy et al. 2003) and visual temporal discrimination thresholds are normal in patients with musician’s dystonia (Maguire et al. 2020) perhaps reflecting better recompensation among elite musicians. Abnormally prolonged sensory temporal discrimination thresholds are present in functional dystonia to the same degree as organic dystonia (Morgante et al. 2011) interpreted as evidence for possible endophenotypic overlap between these disorders. Therapy studies indicate that abnormally prolonged sensory temporal discrimination thresholds in dystonia are not improved by GPi DBS (Sadnicka et al. 2013) or botox therapy (Scontrini et al. 2011). Preserved structural integrity of the sensory system, as determined by somatosensory evoked potentials (SSEPs) may help predict patients more likely to benefit from GPi DBS (McClelland et al. 2018). The effectiveness of sensory trick in cervical dystonia also appears to depend on the remaining integrity of sensory processing, as better visuotactile sensory discrimination correlates with effectiveness of sensory trick (Kägi et al. 2013).

Dystonia, in keeping with current models of a multilevel network disorder, discloses widespread sensory abnormalities, some of which may contribute to development of involuntary movements. The important concept of endophenotype is maintained, as many of the sensory abnormalities may be detected in clinically unaffected body parts or asymptomatic dystonia gene carriers. Future studies are needed to better elucidate environmental triggers and as yet unrecognized intrinsic and external modifying factors. GPi DBS exerts potent inhibition of motor symptoms of dystonia and long-term changes in brainstem and spinal excitability, but seems ineffective in altering sensory endophenotypic defects, which supports their probable upstream role in dystonia pathogenesis.

## Oscillatory activity recordings: local field potentials, microrecordings, EEG and evoked potentials

Recording local oscillatory activity through DBS electrode contacts (local field potentials LFP) can provide some insight into the pathophysiology of dystonia, in particular through the pattern of activity of the basal ganglia-thalamo-cortical pathways. It can also inform us on the mechanisms of action of DBS. Single-cell recordings have been collected during DBS surgery, as part of the process of target localisation, and give us information at unicellular level. Cortical electrical activity recorded through EEG, electrocorticography (EcoG) or magnetic encephalography (MEG) allows to record activity at another point of the basal ganglia-thalamo-cortical circuitry. This activity has often been recorded in conjunction with basal ganglia LFP. Finally evoked potentials (EP) from the DBS pulse within the GPi have been recorded on the cortex.

### Local field potential and cortical recordings

In Parkinson’s disease (PD) many studies have reported increased activity not only in the beta band in the STN but also in GPi (Brown et al. 2001) and a correlation with bradykinesia (Beudel et al. 2017). In dystonia, most recordings have been done in the GPi as it is the preferred DBS target. Several studies have demonstrated an increased power in the low-frequency band (4–12 Hz) in the GPi of dystonic patients (Silberstein et al. 2003; Chen et al. 2006a; Liu et al. 2008; Neumann et al. 2012; Zhu et al. 2018). Correlations between this low-frequency activity and EMG activity have also been shown (Chen et al. 2006a). Using a measure assessing the direction of the coupling, Sharott et al. have shown that the coupling of GPi and the muscles is bidirectional and fluctuating but most of the drive was coming from GPi (Sharott et al. 2008). This low-frequency activity appears to be regulated by peripheral inputs, as demonstrated by a study on ‘geste antagonist’ in cervical dystonia (Tang et al. 2007a). The GPi LFP appear different in dystonia than other conditions such as Huntington disease (Zhu et al. 2018) and suggest that this activity has a role in the expression of the symptoms. LFP power in the 3–21 Hz band appears specific from GPi as it was also shown to be higher than in GPe (Chen et al. 2006b).

The LFP provide also information on the mechanism of action of DBS. The GPi low-frequency activity correlated with the severity of dystonia and a better outcome was observed if the stimulated contact was close to the maximum low-frequency peak (Neumann et al. 2017). A reduction of the mean power in the 4–12 Hz band was observed when DBS was switched on with a decrease in coherence between cortical EEG and GPi LFP in the same band 30 s after switching off DBS (Barow et al. 2014). These changes were observed in patients with phasic dystonia (Barow et al. 2014). Therefore, one of the mechanisms of action of GPi DBS might be through the suppression of abnormally synchronised low-frequency activity between GPi and the cortex.

LFP activity has also been recorded in the STN of dystonic patients, since the STN is also a target for dystonia, although less frequently used (Geng et al. 2017; Neumann et al. 2012). Low-frequency activity was also recorded in the STN in some studies (Geng et al. 2017; Neumann et al. 2012) although Wang et al. (2016) did not find any difference in the STN activity between dystonia and PD patients.

There are many different types of dystonia and not all dystonia would have the same LFP activity. Although GPi DBS can be offered to many different subtypes the outcome tends to be different according to the cause. Many studies have included isolated dystonia, generalised or focal and with or without an identified gene. Two patients with genetically proven myoclonus dystonia patients have also been studied and have shown coherence at 3–15 Hz between GPi and muscles (Foncke et al. 2007) similarly to isolated dystonia. Cervical dystonia has given rise to debate on the role of each GPi according to the pattern of muscle activation and direction of the cervical dystonia. In cervical dystonia interhemispheric differences have been measured in the 4–12 and 13–30 Hz bands (Lee and Kiss 2014). Another study has confirmed a lateralised difference at the level of GPi but not GPe (Moll et al. 2014). Sedov et al. have also found an asymmetry of discharge pattern and Gamma oscillations in 15 patients with cervical dystonia (Sedov et al. 2019a, b).

Most of the LFP recording was performed in the few days after surgery with externalised leads and before battery implantation. Local oedema and lasting effect of anaesthesia could affect those measures. Scheller et al. have recorded nine dystonic patients with long-term DBS using a Medtronic PC + S (Scheller et al. 2019). Patients were assessed with stimulation switched off for 5–7 h, the BFM rating scale was assessed and LFP were recorded. The level of low-frequency activity was associated with the dystonia severity even months after DBS implant and this was specific of that frequency band (Scheller et al. 2019).

It is also important to gain more understanding on the full range of frequency bands and how those activities change during different cognitive and motor tasks. Although some oscillations are probably responsible for the dystonic symptoms, others might still be important for physiological activity. As example it has been demonstrated that walking increased theta-alpha and reduced beta (Singh et al. 2011). Gamma activity has also been shown to change during movement and during cognitive tasks, having a possible role in motor learning (Brücke et al. 2008, 2012; Gillies et al. 2017; Tsang et al. 2012). How DBS affects those physiological frequency changed largely remains to be explored. The role of the low beta (13–21 Hz) cortico-pallidal coherence in initiation and execution of movement has been demonstrated but a lack of correlation with the severity of the dystonia was observed (Van Wijk et al. 2017; Singh et al. 2011; Tsang et al. 2012). Changes in the beta band have also been evidenced in the cortex or cortico-pallidal circuitry (Miocinovic et al. 2015; Neumann et al. 2015; Silberstein et al. 2003). Neumann et al. (2015) identified, with MEG and LFP, three networks: pallido temporal with theta (4–8 Hz) coherence, pallido cerebellar with alpha (7–13 Hz) coherence and cortico pallidal with beta (13–30 Hz) coherence. The last one had a cortical source and the other two a pallidal source. Only the pallido-cerebellar activity was inversely correlated to the severity of the dystonia. A study in 12 patients with generalised or focal isolated dystonia has shown a reduction of the excessive alpha oscillations over the motor cortex and interhemispheric alpha coherence during GPi or STN DBS (Miocinovic et al. 2018). This support the role of network desynchronization in the effect of DBS.

A study in 19 dystonic patients, 10 with phasic dystonia and 9 with tonic dystonia aimed at differentiating activity for these two types of symptoms. In patients with phasic dystonia peaks in the GPi oscillatory activities were observed in the alpha frequency range (8–13 Hz) and was functionally coupled across the GPi, GPe, and the motor cortex. In patients with tonic dystonia, delta oscillatory activities (2–4 Hz) were measured in the GPi with delta GPi–GPe functional coupling (Yokochi et al. 2018).

All the findings related to the role of low-frequency activity in the GPi of dystonia patients have led to the proposal to use this activity as a biomarker for adaptive DBS in dystonia (Neumann et al. 2017; Piña-Fuentes et al. 2019).

### Single-cell recordings

Most studies on micro-recordings have reported that the signal recorded in GPi in patients with dystonia displays irregular grouped discharges with pauses (Vitek et al. 1999; Zhuang et al. 2004; Tang et al. 2007b). Several studies have compared activity in GPi of patients with dystonia and PD. GPi neurons in dystonia have been shown to have significantly lower discharge rates and more irregular discharge patterns than in PD (Vitek et al. 1999; Sanghera et al. 2003; Alam et al. 2016; Tang et al. 2007b; Starr et al. 2005). Higher number of bursts have also been identified in dystonia patients (Alam et al. 2016; Starr et al. 2005). The low-frequency rate has been inversely correlated with the severity of the dystonia (Starr et al. 2005).

Recordings in some studies have been done under general anaesthesia and the role of the anaesthetic agents on the recording is debated. Hutchinson argued that the lower discharge rate recorded in dystonia was an artefact from the use of Propofol (Hutchison et al. 2003). Steigerwald studied the effect of Propofol and concluded that the reduced discharge rate was a real observation (Steigerwald et al. 2005).

Some studies have focussed on the difference between GPi and GPe activity since this information is important for target localisation. In one of the early studies spontaneous discharge rates of GPi and GPe neurons were similar, and the two nuclei were distinguished by neuronal discharge patterns rather than rates (Starr et al. 2006). Sani et al. (2009) recorded pause in the awake human GPe that were characteristic and distinguished primary dystonia from PD and secondary dystonia. Their hypothesis was that they might reflect increased phasic input from striatal D2 receptor positive cells in primary dystonia and are consistent with a recent model proposing that GPe provides capacity scaling for cortical input. Interpause interval (IPI) was lower in primary dystonia (Sani et al. 2009).

Chen et al. (2006a) performed LFP and microrecording in awake dystonic patients to check if the LFP activity comes from the GPi neurones and not from volume conduction. They confirmed that the LFP in the 3–12 Hz band were synchronised to neuronal discharges recorded by microrecordings.

Microrecordings have also been done in the STN of patients implanted with dystonia and compared with PD showing also a lower discharge rate in patients with dystonia. Bursts were observed both in PD and dystonia (Schrock et al. 2009). Zhuang also recorded activity in the STN of dystonic patients and observed reduced rate and irregular bursts (Zhuang et al. 2004).

Devetiarov et al. (2017) have recorded activity on the ventral oral nucleus of the thalamus (Voi) and surrounding areas in patients with cervical dystonia and compared it to activity in the ventro-intermediate nucleus of the thalamus (Vim) in PD patients. Those patients were undergoing thalamotomy to treat their movement disorders. They identified single and burst pattern activities in all areas. They could not identify a disease specific pattern but there was a suggestion that Voa-Vop neurons in the surrounding areas were more hyperpolarised in dystonia because of the inhibitory pallidal outflow (Devetiarov 2017).

### Evoked potentials

Evoked potentials (EP) from the DBS pulses within the GPi have been recorded on the cortex and contribute to our understanding of the mechanism of action of GPi DBS. The studies have demonstrated a peak in the central regions, likely to be the primary motor cortex, around 20–30 ms (Tisch et al. 2008; Bhanpuri et al. 2014; Ni et al. 2018). This peak is larger when the most effective contact is being stimulated and in good responders (Tisch et al. 2008; Bhanpuri et al. 2014). The fact that this peak was absent in a patient who had a previous thalamotomy, combined with the latency, suggest the involvement of the pallido-thalamo-cortical pathway (Tisch et al. 2008). In addition, coherence has been recorded in the beta band (13–30 Hz) between LFP recorded in GPi and motor and premotor oscillatory activity recorded with MEG (Neumann et al. 2015). Ni et al. identified two peaks in the central regions, in addition to the 25 ms peak, they identified an earlier peak at 10 ms (Ni et al. 2018). These two peaks had opposite polarity. The early peak was facilitatory and the later was inhibitory. This supports the hypothesis that the activation of the inhibitory output from GPi leads to inhibition of the motor cortex and normalisation of cortical plasticity in dystonia.

In summary dystonia severity appears related to GPi low frequency activity. One the mechanisms of action of DBS is probably through reducing this activity. This might be used as biomarker for adaptive DBS in the future. Nevertheless, the role of activities in other frequency bands and the effect of DBS on those activities need to be explored further. In addition, we do not know how these findings differ according to the type of dystonia. Microrecordings have confirmed that the low frequency activity recorded with LFP comes from the pallidal neurons and is relevant for the expression of the symptoms including real-time modulation of dystonic contractions by effective sensory tricks. Evoked potentials and recordings of cortical activity also support the role of an inhibition of the motor cortex mediated by the pallido-thalamo-cortical pathway. Finally, adaptive DBS approaches may differ for thalamic targets owing to added complexity of thalamic reorganisation in dystonia (Lenz et al. 1999).

## Conclusions

Dystonia pathophysiology as elucidated by neurophysiology shows wide ranging abnormalities some of which normalise after clinically effective GPi DBS. The keys facts are summarised Table 1. Some changes, for example sensory misprocessing, are likely endophenotypic, providing a substrate for dystonia. Excessive motor cortex plasticity and low frequency pallidal output may play a more direct role in generating dystonic movements. GPi DBS reduces the dystonic symptoms, in proportion to reductions in excessive motor cortex plasticity and low-frequency activity suggesting an important mechanistic role. DBS is not a cure for dystonia and symptoms return after switching DBS off but benefits persist longer in those with more robust plasticity. While GPi DBS has proved a successful and beneficial intervention for dystonia, it has some potential side effects including stimulation-induced Parkinsonian features (Tisch et al. 2007c; Mahlknecht et al. 2018; Kosutzka et al. 2020). Further refinements of DBS including adaptive stimulation may allow improvement in DBS efficacy and side effect profile. Neurophysiology studies will remain essential in furthering our understanding of both dystonia and DBS action.

**Table 1:** Summary of physiology measures abnormalities in dystonia and the effect of GPi DBS

Structure	Technique	Dystonia	Dystonia + GPi DBS
GPi	Single cell	Irregular groups discharges + pauses Lower rate than PD Higher bursts than PD Low frequency correlation severity dystonia	
	LFP	Increased power low frequency band (4–12 Hz) Correlation low frequency—EMG Coupling GPi/muscles bidirectional, drive from GPi Low frequency affected by peripheral input Correlation low frequency severity dystonia Phasic dystonia: alpha (8–13 Hz) coupling GPi, GPe, cortex Tonic dystonia delta (2–4 Hz)	DBS near LF site more effective DBS reduced mean power 4–12 Hz
GPe	Single cells	Different pattern than GPi Pauses different than PD	
	LFP	Lower power in low frequency band than GPi	
STN	Single cell	Lower rate than PD Bursts	
	LFP	Low frequency	
Thalamus (Voi)	Single cell	Single activity and bursts	
Pallido-cortical	LFP	Pallido-temporal theta (4–8 Hz) Pallido-cerebellar alpha (7–13 Hz) inverse correlation severity Cortico-pallidal Beta (13–30 Hz)	DBS reduced cortico-pallidal coherence
	EP from GPi	peak motor cortex 20–30 ms; larger effective contact and if good response (inhibitory) Earlier peak 10 ms (facilitatory)	
Cortex	EEG TMS	Abnormal excessive synchronised 4–12 Hz activity Reduced motor cortex intracortical inhibition and silent period Increased motor cortex plasticity Pre-motor to motor cortex interactions	DBS reduced alpha oscillations motor cortex DBS reduces interhemispheric alpha coherence DBS slightly increases motor cortex excitability DBS over time increases SICI DBS reduces excessive motor cortex plasticity Effects of DBS unknown
Brainstem	Blink reflex	Reduced blink reflex R2 inhibition	DBS normalises R2 disinhibition
Spinal cord	H-reflex	Reduced H-reflex reciprocal inhibition	DBS normalises reduced reciprocal inhibition
Muscle	LFP + EMG	Correlations GPi activity and EMG	

## Abbreviations

DBS: Deep brain stimulation
EcoG: Electrocorticography
EMG: Electromyography
EEG: Electroencephalography
EP: Evoked potentials
GPe: Globus pallidus externus
GPi: Globus pallidus internus
LFP: Local field potentials
LTD: Long-term depression
LTP: Long-term potentiation
MEG: Magnetic encephalography
PAS: Paired associative stimulation
PD: Parkinson’s disease
rTMS: Repetitive transcranial magnetic stimulation
SICI: Short latency intracortical inhibition
SSEPs: Somatosensory evoked potentials
STN: Subthalamic nucleus
TMS: Transcranial magnetic stimulation
Vc: Ventro-caudal nucleus of the thalamus
Vim: Ventro-intermediate nucleus of the thalamus
Voi: Ventral oral nucleus of the thalamus
